# Use of Chloroform in Insanity

**Published:** 1849-01

**Authors:** 


*Use of Chloroform in Insanity.* By Dr. M'Gavin.—The importance of the subject induces us to give entire the following remarks on cases in the Montrose Lunatic Asylum:—The introduction of Chloroform immediately after the nature of this wonderful agent became known, has constituted the only known feature in what may be styled the purely medical part of the treatment. Its beneficial effects have been conspicuous in many cases in which it has been used during the latter part of the last year. The first two patients selected for experiment with the chloroform, were the most noisy and excited in the establishment. One laboured under acute mania; the other was a decided melancholic. In the first case, all the ordinary and approved means for calming excitement and allaying irritability had been, for two or three days, steadily persevered in without much benefit. It then occurred to me that chloroform might be tried. The patient was accordingly secured, which, by the way, was not a very easy task, and the inhalation commenced. The first inspiration was succeeded by a struggle; but this resulted more from dread on the part of the patient that some mischief was to befall him than from any other cause. After a few more inspirations, he complained of sickness; and in less than a minute and a half from the commencement of the inhalation, the functions of the brain were completely suspended. He remained in the comatose state for a minute or two after the withdrawal of the vapour. While recovering from his state of unconsciousness, he looked confused, and reeled about the room like a person under the influence of some intoxicating liquor. In a short time he completely recovered from the immediate effects of the chloroform, but its soothing influence was conspicuous for the greater part of the day. He became drowsy—slept a short time—and was afterwards less excited, and more collected and rational than he had been since his admission. The chloroform was exhibited from time to time in this case, and sleep almost invariably followed. The patient ultimately got better. The second patient operated on was a woman possessed of a strong suicidal tendency, who had been moaning and crying incessantly for days and nights without intermission. She had not been observed to sleep for nearly seventy-two hours. The chloroform was exhibited in the usual way, and very soon reduced her to a state of unconsciousness. On recovering, she complained of sickness, and vomited; after which she was, at her own request, placed in bed, where she enjoyed a sound and refreshing sleep for upwards of three hours. So sensible was this patient of the benefit she derived from the chloroform, that, when afterwards agitated and overwhelmed with despair, she would implore the medical superintendent to repeat the exhibition. In this she was repeatedly indulged. In the first case alluded to, I have very little doubt that the chloroform had a considerable share in conducing to recovery, or at least that it paved the way by suspending the functions of the brain, and thus affording rest to its substance. No doubt the same thing is accomplished in similar cases, and might have followed in this case also, by the exhibition of sedatives and hypnotics; but before sleep is procured in this way, days and weeks sometimes intervene. The rapidity with which the chloroform acts, and the consequent saving of time to the physician, and of nervous energy to the patient, are strong arguments in its favour. It is difficult to say, *a priori*, how far the prolonged exhibition of chloroform may be useful in correcting morbid trains of thought in cases of insanity unattended with excitement. Experiments, with a view to the solution of this question, could not but prove interesting.—*Dublin Medical Press, from Monthly Journal.*

